# An Adaptive Robust Event-Triggered Variational Bayesian Filtering Method with Heavy-Tailed Noise

**DOI:** 10.3390/s25103130

**Published:** 2025-05-15

**Authors:** Di Deng, Peng Yi, Junlin Xiong

**Affiliations:** 1 Department of Control Science and Engineering, Tongji University, Shanghai 201804, China; dengdi@tongji.edu.cn; 2 Shanghai Research Institute for Intelligent Autonomous Systems, Shanghai 201210, China; 3 Department of Automation, University of Science and Technology of China, Hefei 230026, China; xiong77@ustc.edu.cn

**Keywords:** event-triggered scheduling scheme, non-Gaussian noise, robust state estimation, variational Bayesian approach

## Abstract

Event-triggered state estimation has attracted significant attention due to the advantage of efficiently utilizing communication resources in wireless sensor networks. In this paper, an adaptive robust event-triggered variational Bayesian filtering method is designed for heavy-tailed noise with inaccurate nominal covariance matrices. The one-step state prediction probability density function and the measurement likelihood function are modeled as Student’s t-distributions. By choosing inverse Wishart priors, the system state, the prediction error covariance, and the measurement noise covariance are jointly estimated based on the variational Bayesian inference and the fixed-point iteration. In the proposed filtering algorithm, the system states and the unknown covariances are adaptively updated by taking advantage of the event-triggered probabilistic information and the transmitted measurement data in the cases of non-transmission and transmission, respectively. The tracking simulations show that the proposed filtering method achieves good and robust estimation performance with low communication overhead.

## 1. Introduction

State estimation and filtering for dynamic systems has great practical significance in signal processing, communication systems control engineering, and the related fields [1]. State estimation techniques have been widely used in numerous practical applications, such as navigation [2], target tracking [3], and intelligent vehicles [4]. The classical well-known Kalman filter (KF) provides an optimal state estimate for linear Gaussian systems based on the minimum mean squared error (MMSE) criterion [5]. However, the practical systems usually have the phenomenon of incomplete information and non-Gaussian features, which leads to the failure of the Kalman filter.

Constrained by the limited communication resources, the event-triggered scheduling mechanism has emerged as a promising transmission paradigm for optimizing resource utilization while maintaining estimation and control performance by mitigating unnecessary system activations [6,7]. However, incomplete information resulting from the event-triggered scheduling schemes complicates the design of state estimators. Consequently, the design of the event-triggered state estimators has received much attention recently, including the determinist event-triggered scheme [8] and the stochastic event-triggered scheme [9]. Most of the literature on event-triggered state estimation assumes that system noise has a Gaussian noise with known statistical information. This assumption leads to the poor robust estimation performance of the above filters to outliers. As a common type of a non-Gaussian phenomenon, outliers are of enormous practical significance inasmuch as they occur relatively often [10]. The noise corrupted by an outlier has heavy-tailed features, which cannot be captured by a Gaussian distribution [11]. Due to the advantages in dealing with the intractable posterior probability density function (PDF), variational Bayesian inference (VBI) has been an effective approximation method to derive robust estimators.

Motivated by the above discussion, in this paper, we study the robust state estimation with the heavy-tailed process and measurement noise for the stochastic event-triggered scheduling scheme. An adaptive robust event-triggered filtering algorithm is presented based on the VBI approach and the fixed-point iteration. The contributions of this paper are summarized as follows:

1. In the stochastic event-triggered state estimation problem, the heavy-tailed process and measurement noise with inaccurate nominal noise covariances are considered. Student’s t-distribution and the inverse Wishart distribution are adopted to model the one-step prediction and the measurement likelihood PDFs and the unknown covariances matrices, respectively.
2. A robust event-triggered variational Bayesian filtering method is proposed to jointly estimate the system state together with the prediction error covariance and measurement noise covariance. In the filtering algorithm, the event-triggered probabilistic information and the transmitted measurement are, respectively, used to adaptively update the system states in the cases of non-transmission ($\gamma_{k}=0$) and transmission ($\gamma_{k}=1$).
3. The simulation results verify that the proposed filtering method has a promising estimation performance in the presence of intermittent measurements and outliers. The comparison with several other variational filtering algorithms confirms that our filtering greatly saves the communication resources, and achieves a comparable estimation performance without knowing the accurate noise covariances.

The remainder of this paper is organized as follows. Section 2 reviews the literature on event-triggered robust state estimation. Section 3 formulates the stochastic event-triggered robust state estimation problem with the heavy-tailed process and measurement noise, and it presents the hierarchical Gaussian model based on Student’s t-distribution. Section 4 proposes the robust event-triggered variational Bayesian filtering method for cases of non-transmission and transmission, respectively. Section 5 presents the simulation experiments and results to show the effectiveness of the proposed filtering. A conclusion is given in Section 6.

*Notations:* $S_{⪰0}^{n}$ and $S_{≻0}^{n}$ represent the sets of n×n real symmetric positive semi-definite and positive definite matrices, respectively. $I_{n}$ is the n×n-dimensional identity matrix. N(μ,P) denotes the Gaussian distribution with the mean μ and covariance matrix *P*. N(x|μ,P) stands for the Gaussian PDF of random variable *x* with the mean μ and covariance matrix *P*. Pr(·) is the probability of a random event.

## 2. Related Work

Robust state estimation and filtering for dynamic systems have been developing for decades. Robust state estimation has been extensively studied, involving cases of model parameter uncertainty [12], non-Gaussian noise [13], and imperfect sensor information [14,15]. To overcome the failure of conventional Kalman filtering in such problems, various type of filtering methods have been proposed, such as extended Kalman filtering, unscented Kalman filtering, cubature Kalman filtering, and particle filtering.

To date, studies on event-triggered state estimation have produced many exciting results. For the innovation-based deterministic event-triggered scheduling schemes, the authors in [8,16] proposed a Kalman-like filter and a distributed KF by using the Gaussian approximation method, respectively. To obtain the MMSE estimator, a stochastic event-triggered scheduling scheme was proposed in [9] with Gaussian-like triggering functions, and then, the optimal event-triggered KF were derived for both the open-loop and closed-loop schemes. Recently, the robust event-triggered state estimators based on risk-sensitive functions have been proposed for hidden Markov models [17], linear Gaussian systems [18] and nonlinear systems [19], respectively. In [20], an event-triggered model was designed in the presence of packet drops and Gaussian correlated noise. For nonlinear systems, cubature Kalman filtering and particle filtering methods are used in [21,22] to design the event-triggered state estimators, respectively.

Most of the literature on event-triggered state estimation assumes that system noise has a Gaussian distribution with known statistical information. However, in practical engineering applications, many hidden factors and anomalies produce outliers, such as unanticipated environmental disturbances and temporary sensor failure. When the noise is corrupted by outliers, the Kalman-based filters usually have poor robustness because the Gaussian distribution cannot capture heavy-tailed features. In recent years, the filtering problem with non-Gaussian heavy-tailed noise has attracted much attention. Variational Bayesian inference (VBI) has been proposed as an effective approximation method to tackle the complicated PDFs and derive robust filters [23,24,25,26,27]. Based on Student’s t-distribution, a novel variational filtering algorithm with heavy-tailed noise was developed in [23]. In [24], non-stationary heavy-tailed noise is modeled as Gaussian mixture distributions. Lately, the Gaussian-Gamma mixture filter [25], the Gaussian-Student’s t mixture filter [26], and Student’s t mixture filter [27] were proposed in the presence of outliers, respectively. In [28], a multi-sensor variational Bayesian Student’s t-based cubature information fusion algorithm was developed to solve the state estimation of nonlinear systems with heavy-tailed measurement noise. The authors in [29] derived a robust cubature Kalman filter by using a partial variational Bayesian method to deal with non-stationary heavy-tailed noise. For systems with inaccurate Gaussian process noise covariance over binary sensor networks, the authors in [30] developed a distributed sequential estimator via inverse Wishart distributions and fixed-point iterations. In [31], an event-triggered variational Bayesian filter was proposed for the Gaussian system noise with unknown and time-varying noise covariances. In the case of heavy-tailed process noise and Gaussian measurement noise, an event-triggered robust unscented Kalman filter was proposed in [32] to jointly estimate the states and the unmanned surface vehicle parameters. The authors in [33] introduced a sampled memory event-triggered mechanism and discarded the measurement outliers based on an error upper bound.

## 3. Problem Formulation

### 3.1. System Model

Consider the following discrete-time linear system:

(1) $$\begin{array}{c} x_{k}=A_{k}x_{k-1}+w_{k-1}, \end{array}$$

(2) $$\begin{array}{c} y_{k}=C_{k}x_{k}+v_{k}, \end{array}$$

where $x_{k}\in R^{n}$ is the system state, $y_{k}\in R^{m}$ is the measurement output, and the process noise $w_{k}$ and the measurement noise $v_{k}$ are the heavy-tailed noise with zero mean and nominal covariances $Q_{k}\in S_{≻0}^{n}$ and $R_{k}\in S_{≻0}^{m}$, respectively. In practice, both $Q_{k}$ and $R_{k}$ are usually inaccurate. The initial state $x_{0}$ has a Gaussian distribution with mean ${\hat{x}}_{0|0}$ and covariance $P_{0|0}\in S_{≻0}^{n}$, i.e., $p\left(x_{0}\right)=N\left(x_{0}|{\hat{x}}_{0|0},P_{0|0}\right)$. Moreover, $x_{0}$, $w_{k}$, and $v_{k}$ are assumed to be mutually uncorrelated for all $k\in N_{0}$.

To save communication resources for the network, the sensor decides whether to send the measurement to the remote estimator according to the following stochastic event-triggered scheme [9]:

(3) $$\gamma_{k}=\left\{\begin{array}{cc} 1, & \;\mathrm{if}\;\varsigma_{k}>\exp\left(-\frac{1}{2}{\tilde{y}}_{k|k-1}^{⊤}Y{\tilde{y}}_{k|k-1}\right)\\ 0, & \;\mathrm{if}\;\varsigma_{k}\leq \exp\left(-\frac{1}{2}{\tilde{y}}_{k|k-1}^{⊤}Y{\tilde{y}}_{k|k-1}\right), \end{array}\right.$$

where $\gamma_{k}=1$ means transmission; otherwise, no transmission occurs. The innovation ${\tilde{y}}_{k|k-1}$ is defined as ${\tilde{y}}_{k|k-1}=y_{k}-{\hat{y}}_{k|k-1}$ with the feedback prediction measurement ${\hat{y}}_{k|k-1}=C_{k}{\hat{x}}_{k|k-1}$, and the random variable $\varsigma_{k}$ is independent and identically distributed (i.i.d.) following a uniform distribution over [0,1].

At each time *k*, the remote estimator produces a real-time estimate of the system state $x_{k}$ based on the information set

(4) $$I_{k}≜\left\{\gamma_{0},\gamma_{0}y_{0};\ldots ;\gamma_{k},\gamma_{k}y_{k}\right\},\;k\in N_{0}$$

with the initial information set $I_{-1}≜⌀$.

The goal of this paper is to design a robust event-triggered variational Bayesian filtering method based on information set $I_{k}$ for systems (1)–(2) with heavy-tailed process and measurement noise. To this end, Student’s t-distribution, inverse Wishart distribution, and Gamma distribution are first introduced as follows.

**Definition** **1**  ([34])**.** *For a D-dimensional variable x, Student’s t-distribution of variable x is given by*

$$\mathrm{St}\left(x|\mu ,\;\Sigma ,\;\nu \right)\;=\;\frac{\Gamma (\nu /2+D/2)}{\Gamma \left(\nu /2\right){\left(\nu \pi \right)}^{D/2}{\left|\Sigma \right|}^{1/2}}{\left[\;1\;+\;\frac{{\left(\;x-\mu \;\right)}^{⊤}\;\Sigma^{-1}\;\left(\;x-\mu \;\right)}{\nu }\right]}^{-\;\frac{\nu +D}{2}},$$

*where μ is the mean vector, *Σ* is the scale matrix, ν is the degree of freedom (dof), and Γ(·) denotes the Gamma function. The particular case of ν=1 is the Cauchy distribution. In the limit ν→∞, Student’s t-distribution reduces to a Gaussian distribution with the mean μ and covariance *Σ*.*

**Definition** **2**  ([35])**.** *For a p×p-dimensional symmetric positive definite random matrix *Λ*, the inverse Wishart distribution is given by*

$$\mathrm{IW}\left(\Lambda |d,\Psi \right)\;=\;B^{-1}{\;\left(\;d,\;\Psi \;\right)|\Psi |}^{d/2}{\left|\Lambda \right|}^{-(d\;+\;p\;+\;1)/2}\exp\left\{-\mathrm{tr}(\Psi \Lambda^{-1})/2\right\},$$

*where $B\left(d,\Psi \right)=2^{dp/2}\pi^{p(p-1)/4}\Pi_{i=1}^{p}\Gamma \left\{\left(d+1-i\right)/2\right\}$, d is the dof parameter, and *Ψ* is a p×p-dimensional symmetric positive definite inverse scale matrix. If Λ∼IW(Λ|d,Ψ), then $E\left[\Lambda^{-1}\right]=d\Psi^{-1}$ and $E\left[\Lambda \right]={\left(d-p-1\right)}^{-1}\Psi$, provided λ>d+1.*

**Definition** **3**  ([34])**.** *For a positive random variable τ>0 governed by parameters a>0 and b>0, the Gamma distribution of variable τ is given by*

$$G\left(\tau |a,b\right)=\frac{1}{\Gamma (a)}b^{a}\tau^{a-1}e^{-b\tau }.$$

*If τ∼G(τ|a,b), then $E\left[\tau \right]=\frac{a}{b}$.*

**Remark** **1.**  *Compared with the works on event-triggered state estimation [9,36], heavy-tailed noise with inaccurate nominal covariance is considered in the paper. In this case, Kalman-based filters will result in substantial estimation errors because the required Gaussian assumption no longer holds. Hence, the VBI approach is adopted to deal with the non-Gaussianity by choosing appropriate PDFs. Furthermore, the event-triggered scheme leads to incomplete measurements; thus, a way to achieve robust estimation in the case of non-transmission is a critical problem.*

### 3.2. Student’s t-Based Hierarchical Gaussian State-Space Model

In this subsection, Student’s t-based hierarchical Gaussian state-space model is introduced to obtain an approximate of the analytical estimates.

First, due to heavy-tailed noise, the measurement noise $v_{k}$, the likelihood PDF $p(y_{k}|x_{k})$, and the one-step prediction PDF $p(x_{k}|I_{k-1})$ are formulated as Student’s t-distributions as follows [23]:

(5) $$\begin{array}{c} p(v_{k})=\mathrm{St}(v_{k}|0,R_{k},\nu ), \end{array}$$

(6) $$\begin{array}{c} p(y_{k}|x_{k},R_{k})=\mathrm{St}(y_{k}|C_{k}x_{k},R_{k},\nu ), \end{array}$$

(7) $$\begin{array}{c} p(x_{k}|I_{k-1},\Sigma_{k})=\mathrm{St}(x_{k}|{\hat{x}}_{k|k-1},\Sigma_{k},\omega ). \end{array}$$

The prior state estimate ${\hat{x}}_{k|k-1}$ and the nominal prediction error covariance $P_{k|k-1}$ are recursively given by

(8) $$\begin{array}{c} {\hat{x}}_{k|k-1}=A_{k}{\hat{x}}_{k-1|k-1}, \end{array}$$

(9) $$\begin{array}{c} P_{k|k-1}=A_{k}P_{k-1|k-1}A_{k}^{⊤}+Q_{k-1}. \end{array}$$

The covariance $Q_{k-1}$ is not accurate and the heavy-tailed noise $w_{k}$ will result in significant estimation error; hence, variational inference is adopted to estimate the scale matrix $\Sigma_{k}$ instead of $P_{k|k-1}$.

The unknown covariance matrices are usually modeled by inverse Wishart distributions [35]; thus, the conjugate prior distributions of $\Sigma_{k}$ and $R_{k}$ are given as

(10) $$\begin{array}{c} p(\Sigma_{k}|I_{k-1})=\mathrm{IW}(\Sigma_{k}|{\hat{o}}_{k|k-1},{\hat{O}}_{k|k-1}), \end{array}$$

(11) $$\begin{array}{c} p(R_{k}|I_{k-1})=\mathrm{IW}(R_{k}|{\hat{u}}_{k|k-1},{\hat{U}}_{k|k-1}). \end{array}$$

To capture the prior information of $\Sigma_{k}$, the prior parameters ${\hat{o}}_{k|k-1}$ and ${\hat{O}}_{k|k-1}$ satisfy

$$\frac{{\hat{O}}_{k|k-1}}{{\hat{o}}_{k|k-1}}={\tilde{P}}_{k|k-1}=A_{k}P_{k-1|k-1}A_{k}^{⊤}+{\tilde{Q}}_{k-1}$$

where ${\tilde{Q}}_{k-1}$ is the inaccurate nominal process noise covariance. Let ${\hat{o}}_{k|k-1}=\tau$, where τ>0 is a tuning parameter used to reflect the influence of the prior information. It follows that ${\hat{O}}_{k|k-1}=\tau P_{k|k-1}$. The prior parameters ${\hat{u}}_{k|k-1}$ and ${\hat{U}}_{k|k-1}$ are given by [37]

$$\begin{array}{cc} {\hat{u}}_{k|k-1} & =\rho {\hat{u}}_{k-1|k-1},\\ {\hat{U}}_{k|k-1} & =\rho {\hat{U}}_{k-1|k-1}, \end{array}$$

where ρ∈(0,1] is a forgetting factor which indicates the extent of the time fluctuations. The initial $R_{0}$ is also assumed to be inverse Wishart distributed with the dof parameter $u_{0|0}$ and the inverse scale matrix $U_{0|0}$. Then, the initial nominal ${\tilde{R}}_{0}$ is set as $\frac{{\hat{U}}_{0|0}}{{\hat{u}}_{0|0}}={\tilde{R}}_{0}$.

Student’s t-distribution can viewed as an infinite mixture of Gaussians having the same mean but different covariances. Then, $p(x_{k}|I_{k-1})$ and $p(y_{k}|x_{k})$ can be rewritten as [23]

(12) $$\begin{array}{c} p(x_{k}|I_{k-1},\Sigma_{k})=\int N\left(x_{k}|{\hat{x}}_{k|k-1},\Sigma_{k}/\xi_{k}\right)G\left(\xi_{k}|\omega /2,\omega /2\right)d\xi_{k}, \end{array}$$

(13) $$\begin{array}{c} p(y_{k}|x_{k},R_{k})=\int N\left(y_{k}|C_{k}x_{k},R_{k}/\lambda_{k}\right)G\left(\lambda_{k}|\nu /2,\nu /2\right)d\lambda_{k}. \end{array}$$

Therefore, the one-step prediction PDF $p(x_{k}|I_{k-1})$ and the likelihood PDF $p(y_{k}|x_{k})$ are given by the following hierarchical Gaussian forms:

(14) $$\begin{array}{c} p(x_{k}|I_{k-1},\Sigma_{k},\xi_{k})=N(x_{k}|{\hat{x}}_{k|k-1},\Sigma_{k}/\xi_{k}), \end{array}$$

(15) $$\begin{array}{c} p(y_{k}|x_{k},R_{k},\lambda_{k})=N(y_{k}|C_{k}x_{k},R_{k}/\lambda_{k}), \end{array}$$

where variables $\xi_{k}$ and $\lambda_{k}$ obey the following Gamma distributions:

(16) $$\begin{array}{c} p(\xi_{k})=G(\xi_{k}|\omega /2,\omega /2), \end{array}$$

(17) $$\begin{array}{c} p(\lambda_{k})=G(\lambda_{k}|\nu /2,\nu /2). \end{array}$$

As a result, Student’s t-based hierarchical Gaussian state-space model (14)–(17) has been constructed.

## 4. Robust Event-Triggered Variational Bayesian Filtering

In this section, a robust filtering algorithm is proposed based on the VBI approach under event-triggered scheduling scheme (3).

VBI is usually applied to solve the optimization problem for systems with both unknown parameters and latent variables. Denote the set of all latent variables and parameters by $\Omega =\{\theta_{1},\ldots ,\theta_{N}\}$ and the set of all observed variables by $Y=\{y_{1},\ldots ,y_{M}\}$. The solution to the joint posterior PDF p(Ω|Y) is not analytically tractable, which hinders the estimates of the unknown parameters and latent variables. The VBI approach is used to search for an approximate PDF in a factorized form [34,38]:

(18) $$p\left(\Omega |Y\right)\approx \prod_{i=1}^{N}q\left(\theta_{i}\right),$$

where q(·) is the approximate posterior PDF of p(·). The approximation based on VBI can be formed by minimizing the Kullback–Leibler (KL) divergence between the separable approximation and the true posterior distributions. Thus, a general expression for the optimal solution is given by [34,38]

(19) $$\log q^{*}\left(\theta_{i}\right)=E_{\Omega^{-\theta_{i}}}^{*}\left\{\log p\left(\Omega ,Y\right)\right\}+c_{\theta_{i}},$$

where $\Omega^{-\theta_{i}}$ is the set of all elements in Ω except $\theta_{i}$, and $c_{\theta_{i}}$ is a constant independent of $\theta_{i}$. When the optimal variational parameters are coupled with each other, the fixed-point iteration method is adopted to solve (19), i.e.,

(20) $$\log q^{i+1}\left(\theta \right)=E_{\Omega^{-\theta_{i}}}^{i}\left\{\log p\left(\Omega ,Y\right)\right\}+c_{\theta_{i}},$$

where *i* denotes the *i*-th iteration, and the iterations will converge to a local optimum of (19).

**Figure 1:**
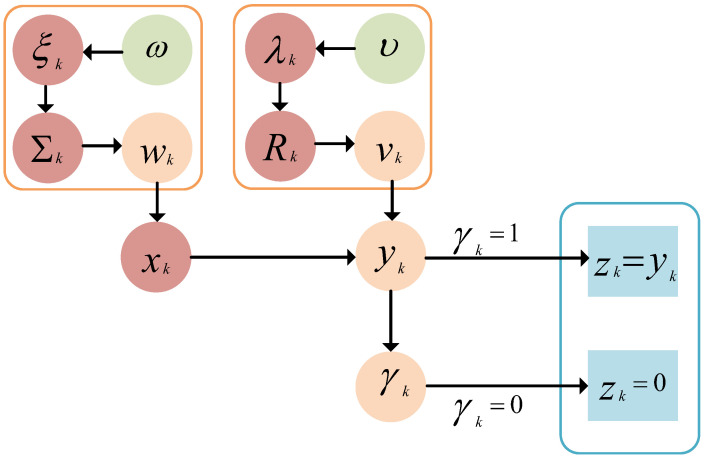
Graphical model for the event-triggered hierarchical Gaussian state-space model based on Student’s t-distribution. The nodes shown with blue rectangles correspond to the observed variable $z_{k}=\gamma_{k}y_{k}$, while the nodes with red circles correspond to the inferred variables $\xi_{k}$, $\lambda_{k}$, $\Sigma_{k}$, $R_{k}$, and $x_{k}$. The blue box represents the two types of observed variables under the event-triggered scheme. The orange boxes represent the latent variables related to the heavy-tailed noise $w_{k}$ and $v_{k}$.

For Student’s t-based hierarchical Gaussian state-space model in Section 3.2, the posterior estimates will be given based on the VBI method, which is illustrated by the graphical model in Figure 1. Depending on whether the measurement data have been received by the remote estimator, the discussion is divided into non-transmission, i.e., $\gamma_{k}=0$, and transmission cases, i.e., $\gamma_{k}=1$. As shown in Figure 1, the measurement $y_{k}$ can be directly used to infer $x_{k}$ in the case of $\gamma_{k}=1$. In the case of $\gamma_{k}=0$, the probabilistic information of the event-triggered scheme (3) is used to infer $x_{k}$ and $y_{k}$ jointly.

**Remark** **2.**  *Compared with most of the previous studies  [23,31], a more practical robust state estimation problem is considered, with both the event-triggered transmission scheme and heavy-tailed noise. A way to estimate the system states in the presence of non-transmissions and outliers is a critical problem. As a result, the proposed filtering will achieve superior robust estimation performance while greatly saving communication resources.*

### 4.1. Variational Filtering in the Case of Non-Transmission

When the measurement $y_{k}$ is not transmitted by the sensor at time *k*, i.e., $\gamma_{k}=0$, $y_{k}$ is unavailable for the remote estimator. In this case, the set of the unknown variables is denoted as

(21) $$\Omega_{0}=\left\{\left(x_{k},y_{k}\right),\xi_{k},\lambda_{k},\Sigma_{k},R_{k}\right\}.$$

In the VBI framework, the joint posterior PDF $p\left(\left(x_{k},y_{k}\right),\xi_{k},\lambda_{k},\Sigma_{k},R_{k}|I_{k}\right)$ is approximated as

(22) $$p\left(\left(x_{k},y_{k}\right),\xi_{k},\lambda_{k},\Sigma_{k},R_{k}|I_{k}\right)\approx q\left(x_{k},y_{k}\right)q\left(\xi_{k}\right)q\left(\lambda_{k}\right)q\left(\Sigma_{k}\right)q\left(R_{k}\right).$$

The approximate posterior PDF for every element in $\Omega_{0}$ will be calculated in the following.

(1) The logarithm of joint PDF $p(\Omega ,I_{k})$: The joint PDF $p(\Omega ,I_{k})$ is factorized as

(23) $$\begin{array}{ccc} p(\Omega_{0},I_{k}) & = & p(\Omega_{0},I_{k-1},\gamma_{k}=0)\\ \; & = & p\left(\gamma_{k}=0|y_{k},I_{k-1}\right)p\left(x_{k},y_{k}|\xi_{k},\lambda_{k},\Sigma_{k},R_{k},I_{k-1}\right)\\ \; & \times & p\left(R_{k}|I_{k-1}\right)p\left(\Sigma_{k}|I_{k-1}\right)p\left(\lambda_{k}\right)p\left(\xi_{k}\right)p\left(I_{k-1}\right), \end{array}$$

where $p(\xi_{k})$, $p(\lambda_{k})$, $p(\Sigma_{k}|I_{k-1})$, and $p(R_{k}|I_{k-1})$ are given by (16), (17), (10) and (11), respectively, and the event-triggered scheme (3) yields

(24) $$p\left(\gamma_{k}=0|y_{k},I_{k-1}\right)=\exp\left[-\frac{1}{2}{\left(y_{k}-{\hat{y}}_{k|k-1}\right)}^{⊤}Y\left(y_{k}-{\hat{y}}_{k|k-1}\right)\right].$$

It has been shown in [9] that $x_{k}$ and $y_{k}$ are jointly Gaussian given $I_{k}$. We define $\phi_{k}≜{\left[x_{k}^{⊤},y_{k}^{⊤}\right]}^{⊤}$, ${\hat{\phi }}_{k|k-1}≜E\left[\phi_{k}|I_{k-1}\right]={\left[{\hat{x}}_{k|k-1}^{⊤},{\hat{y}}_{k|k-1}^{⊤}\right]}^{⊤}$, and $\Phi_{k|k-1}≜E\left[\left(\phi_{k}-{\hat{\phi }}_{k|k-1}\right){\left(\phi_{k}-{\hat{\phi }}_{k|k-1}\right)}^{⊤}\right]$. In view of (12) and (13), $\Phi_{k|k-1}$ is given as

$$\Phi_{k|k-1}=\left[\begin{array}{cc} \Sigma_{k}/\xi_{k} & \Sigma_{k}C_{k}^{⊤}/\xi_{k}\\ C_{k}\Sigma_{k}/\xi_{k} & C_{k}\Sigma_{k}C_{k}^{⊤}/\xi_{k}+R_{k}/\lambda_{k} \end{array}\right].$$

It follows that

$$p\left(x_{k},y_{k}|\xi_{k},\lambda_{k},\Sigma_{k},R_{k},I_{k-1}\right)=N\left(\phi_{k}|{\hat{\phi }}_{k|k-1},\Phi_{k|k-1}\right).$$

Substituting the above distributions into (23), we have

$$\begin{array}{ccc} p(\Omega_{0},I_{k}) & = & \exp\left[-\frac{1}{2}{\left(y_{k}-{\hat{y}}_{k|k-1}\right)}^{⊤}Y\left(y_{k}-{\hat{y}}_{k|k-1}\right)\right]N\left(\phi_{k}|{\hat{\phi }}_{k|k-1},\Phi_{k|k-1}\right)\\ \; & \times & \mathrm{IW}\left(R_{k}|{\hat{u}}_{k|k-1},{\hat{U}}_{k|k-1}\right)\mathrm{IW}\left(\Sigma_{k}|{\hat{o}}_{k|k-1},{\hat{O}}_{k|k-1}\right)\\ \; & \times & G\left(\lambda_{k}|\nu /2,\nu /2\right)G\left(\xi_{k}|\omega /2,\omega /2\right)p\left(I_{k-1}\right). \end{array}$$

Then, $\log p(\Omega_{0},I_{k})$ is computed as

(25) $$\begin{array}{cc} \; & \log p(\Omega_{0},I_{k})\\ = & -\frac{1}{2}{\left(y_{k}-{\hat{y}}_{k|k-1}\right)}^{⊤}Y\left(y_{k}-{\hat{y}}_{k|k-1}\right)-\frac{1}{2}\log{\left|\Phi_{k|k-1}\right|}^{-\frac{1}{2}}\\ \; & -\frac{1}{2}{\left(\phi_{k}-{\hat{\phi }}_{k|k-1}\right)}^{⊤}\Phi_{k|k-1}^{-1}\left(\phi_{k}-{\hat{\phi }}_{k|k-1}\right)\\ \; & -\frac{{\hat{u}}_{k|k-1}+m+1}{2}\log\left|R_{k}\right|-\frac{1}{2}\mathrm{tr}\left({\hat{U}}_{k|k-1}R_{k}^{-1}\right)\\ \; & -\frac{{\hat{o}}_{k|k-1}+n+1}{2}\log\left|\Sigma_{k}\right|-\frac{1}{2}\mathrm{tr}\left({\hat{O}}_{k|k-1}\Sigma_{k}^{-1}\right)\\ \; & +\left(\frac{\omega }{2}-1\right)\log\xi_{k}-\frac{\omega }{2}\xi_{k}+\left(\frac{\nu }{2}-1\right)\log\lambda_{k}-\frac{\nu }{2}\lambda_{k}+C_{\Omega_{0}}, \end{array}$$

where $C_{\Omega_{0}}$ is a constant independent of $\Omega_{0}$.

(2) Decoupling of $\Phi_{k|k-1}$: We used the method proposed in [31] to decouple $\Sigma_{k}$ and $R_{k}$ from $\log|\Phi_{k|k-1}{|}^{-\frac{1}{2}}$ and ${\left(\phi_{k}-{\hat{\phi }}_{k|k-1}\right)}^{⊤}\Phi_{k|k-1}^{-1}\left(\phi_{k}-{\hat{\phi }}_{k|k-1}\right)$. First, $\Phi_{k|k-1}$ is factorized as

$$\Phi_{k|k-1}=\left[\begin{array}{cc} I_{n} & 0\\ C_{k} & I_{m} \end{array}\right]\left[\begin{array}{cc} \Sigma_{k}/\xi_{k} & 0\\ 0 & R_{k}/\lambda_{k} \end{array}\right]\left[\begin{array}{cc} I_{n} & C_{k}^{⊤}\\ 0 & I_{m} \end{array}\right].$$

Thus, the determinant and the inverse of $\Phi_{k|k-1}$ are, respectively, computed as

(26) $$\begin{array}{cc} \; & |\Phi_{k|k-1}\left|=\right|\Sigma_{k}/\xi_{k}\left|\right|R_{k}/\lambda_{k}|=\xi_{k}^{-\frac{n}{2}}\lambda_{k}^{-\frac{m}{2}}|\Sigma_{k}\left|\right|R_{k}|, \end{array}$$

(27) $$\begin{array}{cc} \; & \Phi_{k|k-1}^{-1}=\left[\begin{array}{cc} I_{n} & -C_{k}^{⊤}\\ 0 & I_{m} \end{array}\right]\left[\begin{array}{cc} {\left(\Sigma_{k}/\xi_{k}\right)}^{-1} & 0\\ 0 & {\left(R_{k}/\lambda_{k}\right)}^{-1} \end{array}\right]\left[\begin{array}{cc} I_{n} & 0\\ -C_{k} & I_{m} \end{array}\right]. \end{array}$$

Define $\rho_{k|k-1}=\left[\begin{array}{cc} I_{n} & 0\\ -C_{k} & I_{m} \end{array}\right]\left[\begin{array}{c} x_{k}-{\hat{x}}_{k|k-1}\\ y_{k}-{\hat{y}}_{k|k-1} \end{array}\right]$, then

(28) $$\begin{array}{cc} \; & -\frac{1}{2}{\left(\phi_{k}-{\hat{\phi }}_{k|k-1}\right)}^{⊤}\Phi_{k|k-1}^{-1}\left(\phi_{k}-{\hat{\phi }}_{k|k-1}\right)\\ = & -\frac{1}{2}\rho_{k|k-1}^{⊤}\left[\begin{array}{cc} {\left(\Sigma_{k}/\xi_{k}\right)}^{-1} & 0\\ 0 & {\left(R_{k}/\lambda_{k}\right)}^{-1} \end{array}\right]\rho_{k|k-1}\\ = & -\frac{1}{2}\mathrm{tr}\left(\rho_{k|k-1}\rho_{k|k-1}^{⊤}\left[\begin{array}{cc} {\left(\Sigma_{k}/\xi_{k}\right)}^{-1} & 0\\ 0 & {\left(R_{k}/\lambda_{k}\right)}^{-1} \end{array}\right]\right)\\ = & -\frac{\xi_{k}}{2}\mathrm{tr}\left({\left(\rho_{k|k-1}\rho_{k|k-1}^{⊤}\right)}_{xx}\Sigma_{k}^{-1}\right)-\frac{\lambda_{k}}{2}\mathrm{tr}\left({\left(\rho_{k|k-1}\rho_{k|k-1}^{⊤}\right)}_{yy}R_{k}^{-1}\right), \end{array}$$

where $\rho_{k|k-1}\rho_{k|k-1}^{⊤}$ is computed by

(29) $$\begin{array}{cc} \rho_{k|k-1}\rho_{k|k-1}^{⊤} & =\left[\begin{array}{cc} I_{n} & 0\\ -C_{k} & I_{m} \end{array}\right]\left[\begin{array}{c} x_{k}-{\hat{x}}_{k|k-1}\\ y_{k}-{\hat{y}}_{k|k-1} \end{array}\right]{\left[\begin{array}{c} x_{k}-{\hat{x}}_{k|k-1}\\ y_{k}-{\hat{y}}_{k|k-1} \end{array}\right]}^{⊤}{\left[\begin{array}{cc} I_{n} & 0\\ -C_{k} & I_{m} \end{array}\right]}^{⊤}\\ \; & =\left[\begin{array}{cc} {\left(\rho_{k|k-1}\rho_{k|k-1}^{⊤}\right)}_{xx} & {\left(\rho_{k|k-1}\rho_{k|k-1}^{⊤}\right)}_{xy}\\ {\left(\rho_{k|k-1}\rho_{k|k-1}^{⊤}\right)}_{xy}^{⊤} & {\left(\rho_{k|k-1}\rho_{k|k-1}^{⊤}\right)}_{yy} \end{array}\right], \end{array}$$

and ${\left(\rho_{k|k-1}\rho_{k|k-1}^{⊤}\right)}_{xx}$ and ${\left(\rho_{k|k-1}\rho_{k|k-1}^{⊤}\right)}_{yy}$ are given as

(30) $$\begin{array}{ccc} {\left(\rho_{k|k-1}\rho_{k|k-1}^{⊤}\right)}_{xx} & = & \left(x_{k}-{\hat{x}}_{k|k-1}\right){\left(x_{k}-{\hat{x}}_{k|k-1}\right)}^{⊤},\\ {\left(\rho_{k|k-1}\rho_{k|k-1}^{⊤}\right)}_{yy} & = & C_{k}\left(x_{k}-{\hat{x}}_{k|k-1}\right){\left(x_{k}-{\hat{x}}_{k|k-1}\right)}^{⊤}C_{k}^{⊤}-\left(y_{k}-{\hat{y}}_{k|k-1}\right){\left(x_{k}-{\hat{x}}_{k|k-1}\right)}^{⊤}C_{k}^{⊤}\\ \; & - & C_{k}\left(x_{k}-{\hat{x}}_{k|k-1}\right){\left(y_{k}-{\hat{y}}_{k|k-1}\right)}^{⊤}+\left(y_{k}-{\hat{y}}_{k|k-1}\right){\left(y_{k}-{\hat{y}}_{k|k-1}\right)}^{⊤}. \end{array}$$

Substituting (26) and (28) into (25) yields

(31) $$\begin{array}{cc} \; & \log p(\Omega_{0},I_{k})\\ = & \left(\frac{n+\omega }{2}-1\right)\log\xi_{k}-\frac{\omega }{2}\xi_{k}-\frac{{\hat{o}}_{k|k-1}+n+2}{2}\log\left|\Sigma_{k}\right|\\ \; & -\frac{1}{2}\mathrm{tr}\left({\hat{O}}_{k|k-1}\Sigma_{k}^{-1}\right)-\frac{\xi_{k}}{2}\mathrm{tr}\left({\left(\rho_{k|k-1}\rho_{k|k-1}^{⊤}\right)}_{xx}\Sigma_{k}^{-1}\right)\\ \; & +\left(\frac{m+\nu }{2}-1\right)\log\lambda_{k}-\frac{\nu }{2}\lambda_{k}-\frac{{\hat{u}}_{k|k-1}+m+2}{2}\log\left|R_{k}\right|\\ \; & -\frac{1}{2}\mathrm{tr}\left({\hat{U}}_{k|k-1}R_{k}^{-1}\right)-\frac{\lambda_{k}}{2}\mathrm{tr}\left({\left(\rho_{k|k-1}\rho_{k|k-1}^{⊤}\right)}_{yy}R_{k}^{-1}\right)\\ \; & -\frac{1}{2}{\left(y_{k}-{\hat{y}}_{k|k-1}\right)}^{⊤}Y\left(y_{k}-{\hat{y}}_{k|k-1}\right)+C_{\Omega_{0}}. \end{array}$$

(3) The update of $\xi_{k}$: Let $\theta =\xi_{k}$, and using (31) in (20), we have

(32) $$\log q^{i+1}\left(\xi_{k}\right)=\left(\frac{n+\omega }{2}-1\right)\log\xi_{k}-\frac{1}{2}\xi_{k}\left\{\omega +\mathrm{tr}\left(S_{k}^{i}E^{i}\left[\Sigma_{k}^{-1}\right]\right)\right\}+C_{\xi_{k}},$$

where $S_{k}^{i}$ is given by

(33) $$S_{k}^{i}=E^{i}\left[{\left(\rho_{k|k-1}\rho_{k|k-1}^{⊤}\right)}_{xx}\right].$$

According to (32) and Definition 3, $q^{i+1}\left(\xi_{k}\right)$ can be updated as the following Gamma PDF:

(34) $$q^{i+1}\left(\xi_{k}\right)=G\left(\xi_{k}|\alpha_{k}^{i+1},\beta_{k}^{i+1}\right),$$

where the shape parameter $\alpha_{k}^{i+1}$ and rate parameter $\beta_{k}^{i+1}$ are given by

(35) $$\alpha_{k}^{i+1}=\frac{n+\omega }{2},\;\beta_{k}^{i+1}=\frac{\omega +\mathrm{tr}\left(S_{k}^{i}E^{i}\left[\Sigma_{k}^{-1}\right]\right)}{2}.$$

(4) The update of $\lambda_{k}$: Let $\theta =\lambda_{k}$, and using (31) in (20), we have

(36) $$\log q^{i+1}\left(\lambda_{k}\right)=\left(\frac{m+\nu }{2}-1\right)\log\lambda_{k}-\frac{1}{2}\lambda_{k}\left\{\nu +\mathrm{tr}\left(D_{k}^{i}E^{i}\left[R_{k}^{-1}\right]\right)\right\}+C_{\lambda_{k}},$$

where $D_{k}^{i}$ is given by

(37) $$D_{k}^{i}=E^{i}\left[{\left(\rho_{k|k-1}\rho_{k|k-1}^{⊤}\right)}_{yy}\right].$$

According to (36) and Definition 3, $q^{i+1}\left(\lambda_{k}\right)$ can be updated as the following Gamma PDF:

(38) $$q^{i+1}\left(\lambda_{k}\right)=G\left(\lambda_{k}|\eta_{k}^{i+1},\delta_{k}^{i+1}\right),$$

where the shape parameter $\eta_{k}^{i+1}$ and rate parameter $\delta_{k}^{i+1}$ are given by

(39) $$\eta_{k}^{i+1}=\frac{m+\nu }{2},\;\delta_{k}^{i+1}=\frac{\nu +\mathrm{tr}\left(D_{k}^{i}E^{i}\left[R_{k}^{-1}\right]\right)}{2}.$$

(5) The update of $\Sigma_{k}$: Let $\theta =\Sigma_{k}$, and using (31) in (20), we have

(40) $$\log q^{i+1}\left(\Sigma_{k}\right)=-\frac{{\hat{o}}_{k|k-1}+n+2}{2}\log\left|\Sigma_{k}\right|-\frac{1}{2}\mathrm{tr}\left[\left({\hat{O}}_{k|k-1}+E^{i+1}\left[\xi_{k}\right]S_{k}^{i}\right)\Sigma_{k}^{-1}\right]+C_{\Sigma_{k}}.$$

Based on (40) and Definition 2, $q^{i+1}\left(\Sigma_{k}\right)$ is updated by the following inverse Wishart PDF:

(41) $$q^{i+1}\left(\Sigma_{k}\right)=\mathrm{IW}\left(\Sigma_{k}|{\hat{o}}_{k}^{i+1},{\hat{O}}_{k}^{i+1}\right),$$

where the dof parameter ${\hat{o}}_{k}^{i+1}$ and inverse scale matrix ${\hat{O}}_{k}^{i+1}$ are given by

(42) $${\hat{o}}_{k}^{i+1}={\hat{o}}_{k|k-1}+1,\;{\hat{O}}_{k}^{i+1}={\hat{O}}_{k|k-1}+E^{i+1}\left[\xi_{k}\right]S_{k}^{i}.$$

(6) The update of $R_{k}$: Let $\theta =R_{k}$ and using (31) in (20), we have

(43) $$\begin{array}{cc} \log q^{i+1}\left(R_{k}\right)= & -\frac{{\hat{u}}_{k|k-1}+m+2}{2}\log\left|R_{k}\right|\\ \; & -\frac{1}{2}\mathrm{tr}\left[\left({\hat{U}}_{k|k-1}+E^{i+1}\left[\lambda_{k}\right]D_{k}^{i}\right)R_{k}^{-1}\right]+C_{R_{k}}. \end{array}$$

Based on (43) and Definition 2, $q^{i+1}\left(R_{k}\right)$ can be updated as the following inverse Wishart PDF:

(44) $$q^{i+1}\left(R_{k}\right)=\mathrm{IW}\left(R_{k}|{\hat{u}}_{k}^{i+1},{\hat{U}}_{k}^{i+1}\right),$$

where the dof parameter ${\hat{u}}_{k}^{i+1}$ and inverse scale matrix ${\hat{U}}_{k}^{i+1}$ are given by

(45) $${\hat{u}}_{k}^{i+1}={\hat{u}}_{k|k-1}+1,\;{\hat{U}}_{k}^{i+1}={\hat{U}}_{k|k-1}+E^{i+1}\left[\lambda_{k}\right]D_{k}^{i}.$$

(7) The update of $\phi_{k}$: To update $\phi_{k}$, i.e., $x_{k}$ and $y_{k}$, the following two lemmas will be used.

**Lemma** **1**  ([39])**.** *For matrices A, B, C, and D with appropriate dimensions, if A and $E=D-CA^{-1}B$ are nonsingular, then*

(46) $${\left[\begin{array}{cc} A & B\\ C & D \end{array}\right]}^{-1}=\left[\begin{array}{cc} A^{-1}+A^{-1}BE^{-1}CA^{-1} & -A^{-1}BE^{-1}\\ -E^{-1}CA^{-1} & E^{-1} \end{array}\right],$$

*If D is also nonsingular, then $A^{-1}+A^{-1}BE^{-1}CA^{-1}={\left(A-BD^{-1}C\right)}^{-1}$.*

**Lemma** **2**  ([40])**.** *For matrices A, B, U, and V with appropriate dimensions, if A and B are nonsingular, then ${\left(A+UBV\right)}^{-1}=A^{-1}-A^{-1}U{\left(B^{-1}+VA^{-1}U\right)}^{-1}VA^{-1}$.*

**Theorem** **1.**  *Considering the event-triggered state estimation system with heavy-tailed process and measurement noise (1)–(3), based on Student’s t-based hierarchical Gaussian model (14)–(17) and the variation Bayesian approximation (22), the variational updates ${\hat{x}}_{k|k}^{i+1}$ and ${\hat{y}}_{k|k}^{i+1}$ in the case of $\gamma_{k}=0$ are given as follows:*

(47) $$\begin{array}{cc} {\hat{x}}_{k|k}^{i+1} & ={\hat{x}}_{k|k-1},\;{\hat{y}}_{k|k}^{i+1}=C_{k}{\hat{x}}_{k|k-1}, \end{array}$$

$$\begin{array}{cc} P_{k|k}^{i+1} & ={\tilde{P}}_{k|k-1}^{i+1}-K_{k}^{i+1}C_{k}{\tilde{P}}_{k|k-1}^{i+1}, \end{array}$$

$$\begin{array}{cc} K_{k}^{i+1} & ={\tilde{P}}_{k|k-1}^{i+1}C_{k}^{⊤}{\left(C_{k}{\tilde{P}}_{k|k-1}^{i+1}C_{k}^{⊤}+{\tilde{R}}_{k}^{i+1}+Y^{-1}\right)}^{-1}, \end{array}$$

$$\begin{array}{cc} P_{yy,k|k}^{i+1} & ={\left[{\left({\tilde{R}}_{k}^{i+1}+C_{k}{\tilde{P}}_{k|k-1}^{i+1}C_{k}^{⊤}\right)}^{-1}+Y\right]}^{-1}, \end{array}$$

(48) $$\begin{array}{cc} P_{xy,k|k}^{i+1} & ={\tilde{P}}_{k|k-1}^{i+1}C_{k}^{⊤}{\left[I_{m}+Y\left({\tilde{R}}_{k}^{i+1}+C_{k}{\tilde{P}}_{k|k-1}^{i+1}C_{k}^{⊤}\right)\right]}^{-1}, \end{array}$$

*where*

(49) $${\tilde{R}}_{k}^{i+1}=\frac{{\left(E^{i+1}\left[R_{k}^{-1}\right]\right)}^{-1}}{E^{i+1}\left[\lambda_{k}\right]},\;{\tilde{P}}_{k|k-1}^{i+1}=\frac{{\left(E^{i+1}\left[\Sigma_{k}^{-1}\right]\right)}^{-1}}{E^{i+1}\left[\xi_{k}\right]}.$$

**Proof.**  Let $\theta =\phi_{k}$, and using (31) in (20), we have

(50) $$\begin{array}{cc} \log q^{i+1}\left(\phi_{k}\right)= & -\frac{1}{2}\mathrm{tr}\left(E^{i+1}\left[\xi_{k}\right]{\left(\rho_{k|k-1}\rho_{k|k-1}^{⊤}\right)}_{xx}E^{i+1}\left[\Sigma_{k}^{-1}\right]\right)\\ \; & -\frac{1}{2}\mathrm{tr}\left(E^{i+1}\left[\lambda_{k}\right]{\left(\rho_{k|k-1}\rho_{k|k-1}^{⊤}\right)}_{yy}E^{i+1}\left[R_{k}^{-1}\right]\right)\\ \; & -\frac{1}{2}{\left(y_{k}-{\hat{y}}_{k|k-1}\right)}^{⊤}Y\left(y_{k}-{\hat{y}}_{k|k-1}\right)+C_{\phi_{k}}. \end{array}$$

Defining the modified measurement noise and prediction error covariance matrices ${\tilde{R}}_{k}^{i+1}=\frac{{\left(E^{i+1}\left[R_{k}^{-1}\right]\right)}^{-1}}{E^{i+1}\left[\lambda_{k}\right]}$ and ${\tilde{P}}_{k|k-1}^{i+1}=\frac{{\left(E^{i+1}\left[\Sigma_{k}^{-1}\right]\right)}^{-1}}{E^{i+1}\left[\xi_{k}\right]}$, respectively, then

$$\begin{array}{cc} \; & \log q^{i+1}\left(\phi_{k}\right)\\ = & -\frac{1}{2}\mathrm{tr}\left[{\left(\rho_{k|k-1}\rho_{k|k-1}^{⊤}\right)}_{xx}{\left({\tilde{P}}_{k|k-1}^{i+1}\right)}^{-1}\right]-\frac{1}{2}\mathrm{tr}\left[{\left(\rho_{k|k-1}\rho_{k|k-1}^{⊤}\right)}_{yy}{\left({\tilde{R}}_{k}^{i+1}\right)}^{-1}\right]\\ \; & -\frac{1}{2}{\left(y_{k}-{\hat{y}}_{k|k-1}\right)}^{⊤}Y\left(y_{k}-{\hat{y}}_{k|k-1}\right)+C_{\phi_{k}}\\ = & -\frac{1}{2}\rho_{k|k-1}^{⊤}\left[\begin{array}{cc} {\left({\tilde{P}}_{k|k-1}^{i+1}\right)}^{-1} & 0\\ 0 & {\left({\tilde{R}}_{k}^{i+1}\right)}^{-1} \end{array}\right]\rho_{k|k-1}-\frac{1}{2}{\left(y_{k}-{\hat{y}}_{k|k-1}\right)}^{⊤}Y\left(y_{k}-{\hat{y}}_{k|k-1}\right)+C_{\phi_{k}}\\ = & -\frac{1}{2}{\left(\phi_{k}-{\hat{\phi }}_{k|k-1}\right)}^{⊤}E^{i+1}\left[{\tilde{\Phi }}_{k|k-1}\right]\left(\phi_{k}-{\hat{\phi }}_{k|k-1}\right)-\frac{1}{2}{\left(y_{k}-{\hat{y}}_{k|k-1}\right)}^{⊤}Y\left(y_{k}-{\hat{y}}_{k|k-1}\right)+C_{\phi_{k}}\\ = & -\frac{1}{2}{\left(\phi_{k}-{\hat{\phi }}_{k|k-1}\right)}^{⊤}{\left(\Theta_{k}^{i+1}\right)}^{-1}\left(\phi_{k}-{\hat{\phi }}_{k|k-1}\right)+C_{\phi_{k}}, \end{array}$$

where $E^{i+1}\left[{\tilde{\Phi }}_{k|k-1}\right]$ is given as

(51) $$\begin{array}{cc} E^{i+1}\left[{\tilde{\Phi }}_{k|k-1}\right]\; & =\left[\begin{array}{cc} I_{n} & -C_{k}^{⊤}\\ 0 & I_{m} \end{array}\right]\left[\begin{array}{cc} {\left({\tilde{P}}_{k|k-1}^{i+1}\right)}^{-1} & 0\\ 0 & {\left({\tilde{R}}_{k}^{i+1}\right)}^{-1} \end{array}\right]\left[\begin{array}{cc} I_{n} & 0\\ -C_{k} & I_{m} \end{array}\right]\\ \; & =\left[\begin{array}{cc} {\left({\tilde{P}}_{k|k-1}^{i+1}\right)}^{-1}+C_{k}^{⊤}{\left({\tilde{R}}_{k}^{i+1}\right)}^{-1}C_{k} & -C_{k}^{⊤}{\left({\tilde{R}}_{k}^{i+1}\right)}^{-1}\\ -{\left({\tilde{R}}_{k}^{i+1}\right)}^{-1}C_{k} & {\left({\tilde{R}}_{k}^{i+1}\right)}^{-1}+Y \end{array}\right]. \end{array}$$

and $\Theta_{k}^{i+1}$ is given as

(52) $$\begin{array}{ccc} \Theta_{k}^{i+1} & = & {\left(E^{i+1}\left[{\tilde{\Phi }}_{k|k-1}\right]+\left[\begin{array}{cc} 0 & 0\\ 0 & Y \end{array}\right]\right)}^{-1}\\ \; & = & {\left[\begin{array}{cc} {\left({\tilde{P}}_{k|k-1}^{i+1}\right)}^{-1}+C_{k}^{⊤}{\left({\tilde{R}}_{k}^{i+1}\right)}^{-1}C_{k} & -C_{k}^{⊤}{\left({\tilde{R}}_{k}^{i+1}\right)}^{-1}\\ -{\left({\tilde{R}}_{k}^{i+1}\right)}^{-1}C_{k} & {\left({\tilde{R}}_{k}^{i+1}\right)}^{-1}+Y \end{array}\right]}^{-1},\\ \; & = & \left[\begin{array}{cc} P_{k|k}^{i+1} & P_{xy,k|k}^{i+1}\\ {\left(P_{xy,k|k}^{i+1}\right)}^{⊤} & P_{yy,k|k}^{i+1} \end{array}\right]≜{\left[\begin{array}{cc} A_{\Theta } & B_{\Theta }\\ C_{\Theta } & D_{\Theta } \end{array}\right]}^{-1}. \end{array}$$

Based on Lemma 1, the estimation error covariance of $x_{k}$ is computed as

(53) $$\begin{array}{cc} P_{k|k}^{i+1}= & {\left(A_{\Theta }-B_{\Theta }D_{\Theta }^{-1}C_{\Theta }\right)}^{-1}\\ \; & ={\left[{\left({\tilde{P}}_{k|k-1}^{i+1}\right)}^{-1}+C_{k}^{⊤}\left[{\left({\tilde{R}}_{k}^{i+1}\right)}^{-1}-{\left({\tilde{R}}_{k}^{i+1}\right)}^{-1}{\left({\left({\tilde{R}}_{k}^{i+1}\right)}^{-1}+Y\right)}^{-1}{\left({\tilde{R}}_{k}^{i+1}\right)}^{-1}\right]C_{k}\right]}^{-1}\\ \; & ={\left[{\left({\tilde{P}}_{k|k-1}^{i+1}\right)}^{-1}+C_{k}^{⊤}{\left({\tilde{R}}_{k}^{i+1}+Y^{-1}\right)}^{-1}C_{k}\right]}^{-1}\\ \; & ={\tilde{P}}_{k|k-1}^{i+1}-{\tilde{P}}_{k|k-1}^{i+1}C_{k}^{⊤}{\left(C_{k}{\tilde{P}}_{k|k-1}^{i+1}C_{k}^{⊤}+{\tilde{R}}_{k}^{i+1}+Y^{-1}\right)}^{-1}C_{k}{\tilde{P}}_{k|k-1}^{i+1}\\ \; & ≜{\tilde{P}}_{k|k-1}^{i+1}-K_{k}^{i+1}C_{k}{\tilde{P}}_{k|k-1}^{i+1}, \end{array}$$

where the last two equalities hold because of Lemma 2.

The estimation error covariance of $y_{k}$ is computed as

(54) $$\begin{array}{ccc} P_{yy,k|k}^{i+1} & = & {\left(D_{\Theta }-C_{\Theta }A_{\Theta }^{-1}B_{\Theta }\right)}^{-1}\\ \; & = & [{\left({\tilde{R}}_{k}^{i+1}\right)}^{-1}-{\left({\tilde{R}}_{k}^{i+1}\right)}^{-1}C_{k}({\left({\tilde{P}}_{k|k-1}^{i+1}\right)}^{-1}\\ \; & + & C_{k}^{⊤}{\left({\tilde{R}}_{k}^{i+1}\right)}^{-1}C_{k}{)}^{-1}C_{k}^{⊤}{\left({\tilde{R}}_{k}^{i+1}\right)}^{-1}+Y{]}^{-1}\\ \; & = & \{{\left({\tilde{R}}_{k}^{i+1}\right)}^{-1}-{\left({\tilde{R}}_{k}^{i+1}\right)}^{-1}C_{k}[{\tilde{P}}_{k|k-1}^{i+1}-{\tilde{P}}_{k|k-1}^{i+1}C_{k}^{⊤}({\tilde{R}}_{k}^{i+1}\\ \; & + & C_{k}{\tilde{P}}_{k|k-1}^{i+1}C_{k}^{⊤}{)}^{-1}C_{k}^{⊤}{\tilde{P}}_{k|k-1}^{i+1}]C_{k}^{⊤}{\left({\tilde{R}}_{k}^{i+1}\right)}^{-1}+Y{\}}^{-1}\\ \; & = & {\left\{{\left({\tilde{R}}_{k}^{i+1}\right)}^{-1}-{\left[{\left(C_{k}{\tilde{P}}_{k|k-1}^{i+1}C_{k}^{⊤}\right)}^{-1}{\tilde{R}}_{k}^{i+1}+I_{m}\right]}^{-1}{\left({\tilde{R}}_{k}^{i+1}\right)}^{-1}+Y\right\}}^{-1}\\ \; & = & {\left[{\left({\tilde{R}}_{k}^{i+1}+C_{k}{\tilde{P}}_{k|k-1}^{i+1}C_{k}^{⊤}\right)}^{-1}+Y\right]}^{-1}, \end{array}$$

where the third equality holds due to Lemma 2, and the last two equalities are obtained by conventional matrix operations.

In addition, the cross-covariance between $x_{k}$ and $y_{k}$ are derived as follows:

(55) $$\begin{array}{ccc} P_{xy,k|k}^{i+1} & = & -A_{\Theta }^{-1}C_{\Theta }P_{yy,k|k}^{i+1}\\ \; & = & {\left[{\left({\tilde{P}}_{k|k-1}^{i+1}\right)}^{-1}+C_{k}^{⊤}{\left({\tilde{R}}_{k}^{i+1}+Y^{-1}\right)}^{-1}C_{k}\right]}^{-1}C_{k}^{⊤}{\left({\tilde{R}}_{k}^{i+1}\right)}^{-1}\\ \; & \times & {\left[{\left({\tilde{R}}_{k}^{i+1}+C_{k}{\tilde{P}}_{k|k-1}^{i+1}C_{k}^{⊤}\right)}^{-1}+Y\right]}^{-1}\\ \; & = & \left[{\tilde{P}}_{k|k-1}^{i+1}-{\tilde{P}}_{k|k-1}^{i+1}C_{k}^{⊤}{\left(C_{k}{\tilde{P}}_{k|k-1}^{i+1}C_{k}^{⊤}+{\tilde{R}}_{k}^{i+1}\right)}^{-1}C_{k}{\tilde{P}}_{k|k-1}^{i+1}\right]\\ \; & \times & C_{k}^{⊤}{\left({\tilde{R}}_{k}^{i+1}\right)}^{-1}{\left[{\left({\tilde{R}}_{k}^{i+1}+C_{k}{\tilde{P}}_{k|k-1}^{i+1}C_{k}^{⊤}\right)}^{-1}+Y\right]}^{-1}\\ \; & = & {\tilde{P}}_{k|k-1}^{i+1}C_{k}^{⊤}{\left({\tilde{R}}_{k}^{i+1}+C_{k}{\tilde{P}}_{k|k-1}^{i+1}C_{k}^{⊤}\right)}^{-1}{\left[{\left({\tilde{R}}_{k}^{i+1}+C_{k}{\tilde{P}}_{k|k-1}^{i+1}C_{k}^{⊤}\right)}^{-1}+Y\right]}^{-1}\\ \; & = & {\tilde{P}}_{k|k-1}^{i+1}C_{k}^{⊤}{\left[I_{m}+Y\left({\tilde{R}}_{k}^{i+1}+C_{k}{\tilde{P}}_{k|k-1}^{i+1}C_{k}^{⊤}\right)\right]}^{-1} \end{array}$$

where the third equality holds due to Lemma 2.

Therefore, $q^{i+1}\left(x_{k}\right)$ is updated as the Gaussian PDF $q^{i+1}\left(x_{k}\right)=N\left(x_{k}|{\hat{x}}_{k|k}^{i+1},P_{k|k}^{i+1}\right)$, where the mean ${\hat{x}}_{k|k}^{i+1}={\hat{x}}_{k|k-1}$ and the covariance matrix $P_{k|k}^{i+1}$ is given as (48). Similarly, $q^{i+1}\left(y_{k}\right)$ is updated as the Gaussian PDF $q^{i+1}\left(y_{k}\right)=N\left(y_{k}|{\hat{y}}_{k|k}^{i+1},P_{yy,k|k}^{i+1}\right)$, where the mean ${\hat{y}}_{k|k}^{i+1}={\hat{y}}_{k|k-1}=C_{k}{\hat{x}}_{k|k-1}$ and the covariance matrix $P_{yy,k|k}^{i+1}$ is given as (48).    □

(8) The computation of expectations: According to Definition 2 and Definition 3, the required expectations $E^{i+1}\left[\xi_{k}\right]$, $E^{i+1}\left[\lambda_{k}\right]$, $E^{i+1}\left[\Sigma_{k}^{-1}\right]$ and $E^{i+1}\left[R_{k}^{-1}\right]$ are calculated as follows

(56) $$\begin{array}{cc} E^{i+1}\left[\xi_{k}\right] & =\alpha_{k}^{i+1}/\beta_{k}^{i+1},\;E^{i+1}\left[\lambda_{k}\right]=\eta_{k}^{i+1}/\delta_{k}^{i+1}, \end{array}$$

(57) $$\begin{array}{cc} E^{i+1}\left[\Sigma_{k}^{-1}\right] & ={\hat{o}}_{k}^{i+1}{\left(\;{\hat{O}}_{k}^{i+1}\;\right)}^{-1},\;E^{i+1}\left[R_{k}^{-1}\right]={\hat{u}}_{k}^{i+1}{\left(\;{\hat{U}}_{k}^{i+1}\;\right)}^{-1}. \end{array}$$

In view of (30), the required expectations $S_{k}^{i+1}$ and $D_{k}^{i+1}$ are computed as

(58) $$\begin{array}{cc} S_{k}^{i+1}= & E^{i+1}{\left[(\rho_{k|k-1}\rho_{k|k-1}^{⊤}\right)}_{xx}]\\ = & P_{k|k}^{i+1}+\left({\hat{x}}_{k|k}^{i+1}-{\hat{x}}_{k|k-1}\right){\left({\hat{x}}_{k|k}^{i+1}-{\hat{x}}_{k|k-1}\right)}^{⊤}, \end{array}$$

(59) $$\begin{array}{cc} D_{k}^{i+1}= & E^{i+1}{\left[(\rho_{k|k-1}\rho_{k|k-1}^{⊤}\right)}_{yy}]\\ = & C_{k}P_{k|k}^{i+1}C_{k}^{⊤}\;-\;{\left(\;C_{k}P_{xy,k|k}^{i+1}\;\right)}^{⊤}\;-\;\left(\;C_{k}P_{xy,k|k}^{i+1}\;\right)\;+\;P_{yy,k|k}^{i+1}, \end{array}$$

where $P_{k|k}^{i+1}$, $P_{yy,k|k}^{i+1}$, and $P_{xy,k|k}^{i+1}$ are given by (48). In the case of $\gamma_{k}=0$, it follows from ${\hat{x}}_{k|k}^{i+1}={\hat{x}}_{k|k-1}$ and $S_{k}^{i}=P_{k|k}^{i}$.

### 4.2. Variational Bayesian Filtering in the Case of Transmission

When the measurement $y_{k}$ is transmitted by the sensor at time *k*, i.e., $\gamma_{k}=1$, the set of the unknown variables is denoted as

(60) $$\Omega_{1}=\left\{x_{k},\xi_{k},\lambda_{k},\Sigma_{k},R_{k}\right\}.$$

The joint PDF $p(\Omega_{1},I_{k})$ is factorized as

(61) $$\begin{array}{cc} \; & p\left(\Omega_{1},I_{k}\right)=p\left(\Omega_{1},I_{k-1},\gamma_{k}=1,y_{k}\right)\\ = & p\left(\gamma_{k}=1|y_{k},I_{k-1}\right)p\left(y_{k}|x_{k},\lambda_{k},R_{k}\right)p\left(x_{k}|\xi_{k},\Sigma_{k},I_{k-1}\right)\\ \; & \times p\left(R_{k}|I_{k-1}\right)p\left(\Sigma_{k}|I_{k-1}\right)p\left(\lambda_{k}\right)p\left(\xi_{k}\right)p\left(I_{k-1}\right)\\ = & \left[1-\exp\left(-\frac{1}{2}{\tilde{y}}_{k|k-1}^{⊤}Y{\tilde{y}}_{k|k-1}\right)\right]N\left(y_{k}|C_{k}x_{k},R_{k}/\lambda_{k}\right)\\ \; & \times N\left(x_{k}|{\hat{x}}_{k|k-1},\Sigma_{k}/\xi_{k}\right)\mathrm{IW}\left(R_{k}|{\hat{u}}_{k-1|k-1},{\hat{U}}_{k-1|k-1}\right)\\ \; & \times \mathrm{IW}\left(\Sigma_{k}|{\hat{o}}_{k-1|k-1},{\hat{O}}_{k-1|k-1}\right)G\left(\lambda_{k}|\nu /2,\nu /2\right)G\left(\xi_{k}|\omega /2,\omega /2\right)p\left(I_{k-1}\right) \end{array}$$

The recursions of $\xi_{k}$, $\lambda_{k}$, $\Sigma_{k}$, and $R_{k}$ are the same as those in the case of $\gamma_{k}=0$. Let $\theta =x_{k}$, so that

(62) $$\begin{array}{cc} \; & \log q^{i+1}\left(x_{k}\right)\\ = & -\frac{1}{2}E^{i+1}\left[\xi_{k}\right]\left(x_{k}-{\hat{x}}_{k|k-1}\right)E^{i+1}\left[\Sigma_{k}^{-1}\right]{\left(x_{k}-{\hat{x}}_{k|k-1}\right)}^{⊤}\\ \; & -\frac{1}{2}E^{i+1}\left[\lambda_{k}\right]\left(y_{k}-C_{k}x_{k}\right)E^{i+1}\left[R_{k}^{-1}\right]{\left(y_{k}-C_{k}x_{k}\right)}^{⊤}+C_{x_{k}}\\ = & -\frac{1}{2}\left(x_{k}-{\hat{x}}_{k|k-1}\right){\left({\tilde{P}}_{k|k-1}^{i+1}\right)}^{-1}{\left(x_{k}-{\hat{x}}_{k|k-1}\right)}^{⊤}\\ \; & -\frac{1}{2}\left(y_{k}-C_{k}x_{k}\right){\left({\tilde{R}}_{k}^{i+1}\right)}^{-1}{\left(y_{k}-C_{k}x_{k}\right)}^{⊤}+C_{x_{k}}, \end{array}$$

where ${\tilde{R}}_{k}^{i+1}$ and ${\tilde{P}}_{k|k-1}^{i+1}$ are given by (49). Hence $x_{k}$ is updated by the following Gaussian PDF:

(63) $$q^{i+1}\left(x_{k}\right)=N\left(x_{k}|{\hat{x}}_{k|k}^{i+1},P_{k|k}^{i+1}\right),$$

where the mean ${\hat{x}}_{k|k}^{i+1}$ and covariance matrix $P_{k|k}^{i+1}$ are given by

(64) $$\begin{array}{cc} {\hat{x}}_{k|k}^{i+1} & ={\hat{x}}_{k|k-1}+K_{k}^{i+1}\left(y_{k}-C_{k}{\hat{x}}_{k|k-1}\right),\\ P_{k|k}^{i+1} & ={\tilde{P}}_{k|k-1}^{i+1}-K_{k}^{i+1}C_{k}{\tilde{P}}_{k|k-1}^{i+1},\\ K_{k}^{i+1} & ={\tilde{P}}_{k|k-1}^{i+1}C_{k}^{⊤}{\left(C_{k}{\tilde{P}}_{k|k-1}^{i+1}C_{k}^{⊤}+{\tilde{R}}_{k}^{i+1}\right)}^{-1}. \end{array}$$

The expectations $S_{k}^{i+1}$ and $D_{k}^{i+1}$ are given as follows.

(65) $$\begin{array}{cc} S_{k}^{i+1}= & P_{k|k}^{i+1}+\left({\hat{x}}_{k|k}^{i+1}-{\hat{x}}_{k|k-1}\right){\left({\hat{x}}_{k|k}^{i+1}-{\hat{x}}_{k|k-1}\right)}^{⊤}, \end{array}$$

(66) $$\begin{array}{cc} D_{k}^{i+1}= & E^{i+1}\left[\left(y_{k}-C_{k}x_{k}\right){\left(y_{k}-C_{k}x_{k}\right)}^{⊤}\right]\\ = & \left(y_{k}-C_{k}{\hat{x}}_{k|k}^{i+1}\right){\left(y_{k}-C_{k}{\hat{x}}_{k|k}^{i+1}\right)}^{⊤}+C_{k}P_{k|k}^{i+1}C_{k}^{⊤}, \end{array}$$

where $P_{k|k}^{i+1}$ is given by (64).

**Remark** **3.**  *In the derivation of variational Bayesian filtering, event-triggered structure information is used to calculate the logarithm of joint PDF $p(\Omega ,I_{k})$ in the case of non-transmission. Despite the absence of the measurement, the unknown covariances are adaptively estimated by taking advantage of the conditional probabilities of the triggering decision (24). In the case of transmission, the measurement received by the remote estimator can be directly used to update the system states, as seen in (64).*

To sum up, we propose the robust event-triggered Student’s t-based variational Bayesian filtering, as summarized in Algorithm 1.
**Algorithm 1** Event-triggered Student’s t-based robust variational Bayesian filtering**Input:** ${\hat{x}}_{k-1|k-1}$, $P_{k-1|k-1}$, $A_{k}$, $C_{k}$, ${\tilde{Q}}_{k-1}$, ${\hat{u}}_{k-1|k-1}$, ${\hat{U}}_{k-1|k-1}$; *m*, *n*, ω, ν, τ, ρ, *N*1:**Time update:**2:${\hat{x}}_{k|k-1}=A_{k}{\hat{x}}_{k-1|k-1}$3:${\tilde{P}}_{k|k-1}=A_{k}P_{k-1|k-1}A_{k}^{⊤}+{\tilde{Q}}_{k-1}$4:**Variational measurement update:**5:Initialization: ${\hat{x}}_{k|k}^{0}={\hat{x}}_{k|k-1}$, $P_{k|k}^{0}={\tilde{P}}_{k|k-1}$, ${\hat{o}}_{k|k-1}=\tau$, ${\hat{O}}_{k|k-1}=\tau {\tilde{P}}_{k|k-1}$, ${\hat{u}}_{k|k-1}=\rho {\hat{u}}_{k-1|k-1}$, ${\hat{U}}_{k|k-1}=\rho {\hat{U}}_{k-1|k-1}$, $E^{0}\left[\Sigma_{k}^{-1}\right]={\hat{o}}_{k|k-1}{\hat{O}}_{k|k-1}^{-1}$, $E^{0}\left[R_{k}^{-1}\right]={\hat{u}}_{k|k-1}{\hat{U}}_{k|k-1}^{-1}$6:**for** i=0 to N−1 **do**7:     **Calculate** $S_{k}^{i}$ and $D_{k}^{i}$:8:     **if** $\gamma_{k}=0$ **then**9:          $S_{k}^{i}=P_{k|k}^{i}$, $D_{k}^{i}$ is calculated by (59)10:     **else**11:          $S_{k}^{i}$ and $D_{k}^{i}$ are calculated by (65) and (66)12:     **end if**13:     **Update** $q^{i+1}\left(\xi_{k}\right)=G\left(\xi_{k}|\alpha_{k}^{i+1},\beta_{k}^{i+1}\right)$ by (35), $E^{i+1}\left[\xi_{k}\right]=\alpha_{k}^{i+1}/\beta_{k}^{i+1}$14:     **Update** $q^{i+1}\left(\lambda_{k}\right)=G\left(\lambda_{k}|\eta_{k}^{i+1},\delta_{k}^{i+1}\right)$ by (39), $E^{i+1}\left[\lambda_{k}\right]=\eta_{k}^{i+1}/\delta_{k}^{i+1}$15:     **Update** $q^{i+1}\left(\Sigma_{k}\right)=\mathrm{IW}\left(\Sigma_{k}|{\hat{o}}_{k}^{i+1},{\hat{O}}_{k}^{i+1}\right)$ by (42), $E^{i+1}\left[\Sigma_{k}^{-1}\right]={\hat{o}}_{k}^{i+1}{\left(\;{\hat{O}}_{k}^{i+1}\;\right)}^{-1}$16:     **Update** $q^{i+1}\left(R_{k}\right)=\mathrm{IW}\left(R_{k}|{\hat{u}}_{k}^{i+1},{\hat{U}}_{k}^{i+1}\right)$ by (45), $E^{i+1}\left[R_{k}^{-1}\right]={\hat{u}}_{k}^{i+1}{\left(\;{\hat{U}}_{k}^{i+1}\;\right)}^{-1}$17:     **Update** $q^{i+1}\left(x_{k}\right)=N\left(x_{k}|{\hat{x}}_{k|k}^{i+1},P_{k|k}^{i+1}\right)$: ${\tilde{R}}_{k}^{i+1}=\frac{{\left(E^{i+1}\left[R_{k}^{-1}\right]\right)}^{-1}}{E^{i+1}\left[\lambda_{k}\right]}$, ${\tilde{P}}_{k|k-1}^{i+1}=\frac{{\left(E^{i+1}\left[\Sigma_{k}^{-1}\right]\right)}^{-1}}{E^{i+1}\left[\xi_{k}\right]}$18:     **if** $\gamma_{k}=0$ **then**19:          ${\hat{x}}_{k|k}^{i+1}={\hat{x}}_{k|k-1}$, $P_{k|k}^{i+1}$, $P_{yy,k|k}^{i+1}$, $P_{xy,k|k}^{i+1}$ are calculated as (48)20:     **else**21:          ${\hat{x}}_{k|k}^{i+1}$ and $P_{k|k}^{i+1}$ are updated as (64)22:     **end if**23:**end for**24:${\hat{x}}_{k|k}={\hat{x}}_{k|k}^{N}$, $P_{k|k}=P_{k|k}^{N}$, ${\hat{o}}_{k|k}={\hat{o}}_{k}^{N}$, ${\hat{O}}_{k|k}={\hat{O}}_{k}^{N}$, ${\hat{u}}_{k|k}={\hat{u}}_{k}^{N}$, ${\hat{U}}_{k|k}={\hat{U}}_{k}^{N}$**Output:** ${\hat{x}}_{k|k}$, $P_{k|k}$, ${\hat{u}}_{k|k}$, ${\hat{U}}_{k|k}$

The parameters in Algorithm 1 are discussed as follows: (1) ν and ω are the dofs of Student’s t-distributions (6) and (7), respectively. Student’s t-distribution will reduce to the Cauchy distribution and the Gaussian distribution when the dof is 1 and infinite, respectively. The selection of the dofs is related to the degree of heavy tail of the target distribution. (2) τ>0 is a tuning parameter used to reflect the influence of the prior information $P_{k|k-1}$. For a large τ, the measurement updates will introduce more prior uncertainties caused by the heavy-tailed process noise. For a small τ, a large amount of information about the system process will be lost. Generally, the tuning parameter is suggested to be selected as τ∈[2,6]. (3) ρ∈(0,1] is the forgetting factor used to update the prior scale matrix in the inverse Wishart prior distribution of $R_{k}$ in (11), which indicates the extent of the time fluctuations. The smaller the forgetting factor ρ, the more the information from the previous estimation ${\hat{R}}_{k-1}$ is forgotten; otherwise, the more the information from the previous estimation ${\hat{R}}_{k-1}$ is used. Generally, the forgetting factor is suggested to be selected as ρ∈[0.94,1] for a better performance.

**Remark** **4.**  *The computational complexity of Algorithm 1 is analyzed. In the time update step, the computational complexity of calculating ${\hat{x}}_{k|k-1}$ and ${\tilde{P}}_{k|k-1}$ is $O(n^{3})$. In the variational measurement update for each iteration i, the complexity of calculating $S_{k}^{i}$ and $D_{k}^{i}$ is $O(n^{2})$ and $O(mn^{2}+nm^{2})$, respectively. The complexity of updating $\xi_{k}$, $\lambda_{k}$, $\Sigma_{k}$, and $R_{k}$ is $O(n^{3})$, $O(m^{3})$, $O(n^{2})$, and $O(m^{2})$, respectively. Then, the computational complexity of obtaining ${\hat{x}}_{k|k}^{i+1}$ is $O(mn^{2}+nm^{2}+m^{3}+n^{3})$, where n and m are the dimensions of the system state and the measurement output, respectively.*

## 5. Simulation Results

In this section, the performance of the proposed robust event-triggered variational Bayesian filtering method is illustrated by the problem of target tracking. The target moves according to a constant velocity model in two-dimensional space, and its position is measured. The system matrices are given as

$$\begin{array}{cc} A_{k}=\left[\begin{array}{cc} I_{2} & TI_{2}\\ 0 & I_{2} \end{array}\right], & C_{k}=\left[\begin{array}{cc} I_{2} & 0 \end{array}\right], \end{array}$$

where the parameter T=1 s is the sampling interval. The state dimension and the measurement dimension are n=4 and m=2, respectively. The nominal process and measurement noise covariances are set as

$$\begin{array}{cc} Q_{k}=\left[\begin{array}{cc} \frac{T^{3}}{3}I_{2} & \frac{T^{2}}{2}I_{2}\\ \frac{T^{2}}{2}I_{2} & TI_{2} \end{array}\right]q,\; & R_{k}=rI_{2}, \end{array}$$

where q=1 and $r=100\;m^{2}$. The heavy-tailed process and measurement noise are generated by

$$w_{k}∼\left\{\begin{array}{cc} N(0,Q_{k}), & w.p.0.95\\ N(0,100Q_{k}), & w.p.0.05, \end{array}\right.\;v_{k}∼\left\{\begin{array}{cc} N(0,R_{k}), & w.p.0.90\\ N(0,100R_{k}), & w.p.0.10, \end{array}\right.$$

In this simulation, we compare the proposed filtering algorithm, Algorithm 1, with the following estimators.

1. RST-KF (the robust Student’s t-based Kalman filter [23]): RST-KF is proposed for heavy-tailed process and measurement noise, where the accurate noise covariances are known and $y_{k}$ is sent at each time *k*.
2. ETVBF (the event-triggered variational Bayesian filter [31]): ETVBF is proposed for unknown Gaussian noise covariance, where the unknown Gaussian process noise covariance is formulated as the multiple nominal process noise covariance.
3. KFNCM (the Kalman filter with nominal noise covariance matrices): The measurement $y_{k}$ is sent at each time *k*.
4. CLSET-KF (the closed-loop stochastic event-triggered Kalman filter [9]): The stochastic event-triggered scheme is adopted, where the process and measurement noise have Gaussian distributions with known covariance matrices.

The inaccurate nominal initial process and measurement noise covariances are set as ${\tilde{Q}}_{k}=I_{4}$ and ${\tilde{R}}_{0}=\beta I_{2}$, respectively. The triggering parameter takes the form of $Y=\zeta I_{2}$. The mean and covariance of $x_{0}$ are chosen as ${\hat{x}}_{0|0}={\left[10,10,10,10\right]}^{⊤}$ and $P_{0|0}=100I_{4}$. The dof parameters are set as ω=ν=5. The tuning parameter and the forgetting factor are set as τ=5 and ρ=0.997, respectively. To compare the estimation performance, the root mean square error (RMSE) and the averaged RMSE (ARMSE) are used as the performance metrics, which are defined as

$$\begin{array}{cc} {\mathrm{RMSE}}_{j,k,l}\;≜\; & {\left[\frac{1}{Mn}\;\sum_{j=1}^{M}\;\sum_{l=1}^{n}{\left(x_{l,k}\left(j\right)\;-\;{\hat{x}}_{l,k|k}\left(j\right)\right)}^{2}\right]}^{\frac{1}{2}},\\ {\mathrm{ARMSE}}_{j,k,l}\;≜\; & {\left[\;\frac{1}{MKn}\;\sum_{j=1}^{M}\;\sum_{k=1}^{K}\;\sum_{l=1}^{n}{\left(x_{l,k}\left(j\right)\;-\;{\hat{x}}_{l,k|k}\left(j\right)\right)}^{2}\;\right]}^{\frac{1}{2}}, \end{array}$$

where M= 10,000, K=200, and *n* represent the total Monte Carlo experiment number, the iterative step of one Monte Carlo run, and the dimension of the state, respectively. $x_{l,k}\left(j\right)$ and ${\hat{x}}_{l,k|k}\left(j\right)$ represent the *l*-th components of system state $x_{k}$ and the estimate ${\hat{x}}_{k|k}$ at time *k* in the *j*-th Monte Carlo run, respectively. To evaluate the estimation performance under different transmission frequencies, the communication rate is defined as

$$\gamma ≜\underset{N\to \infty }{lim sup}\frac{1}{N}\sum_{k=0}^{N-1}E\left[\gamma_{k}\right],$$

which is numerically calculated by

$$\gamma ={\left[\frac{1}{MK}\sum_{j=1}^{M}\sum_{k=1}^{K}\gamma_{k,j}\right]}^{\frac{1}{2}},$$

where $\gamma_{k,j}$ denotes $\gamma_{k}$ in the *j*-th Monte Carlo run.

**Figure 2:**
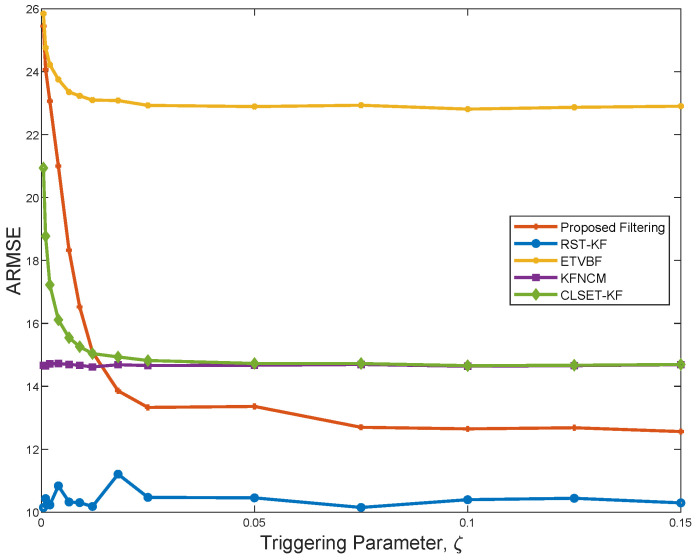
ARMSEs of the proposed filtering, RST-KF, ETVBF, KFNCM, and CLSET-KF versus the triggering parameter.

Figure 2 depicts the ARMSEs of the five filtering algorithms under β=100. It is seen that the proposed filter performs better than ETVBF, KFNCM, and CLSET-KF, and it gradually stabilizes as ζ increases. Compared with RST-KF, the proposed filter does not know the exact nominal noise covariance. Hence, the performance of the proposed filter is worse than that of RST-KF because of the information loss. ETVBF is developed for the unknown Gaussian noise covariances, and it has poor estimation performance in the presence of outliers. KFNCM and CLSET-KF are the optimal estimators for the linear Gaussian systems without and with closed-loop the event-triggered scheme, respectively, and their performance tends to be the same as ζ increases. However, the two filters are ineffective under heavy-tailed noise.

**Figure 3:**
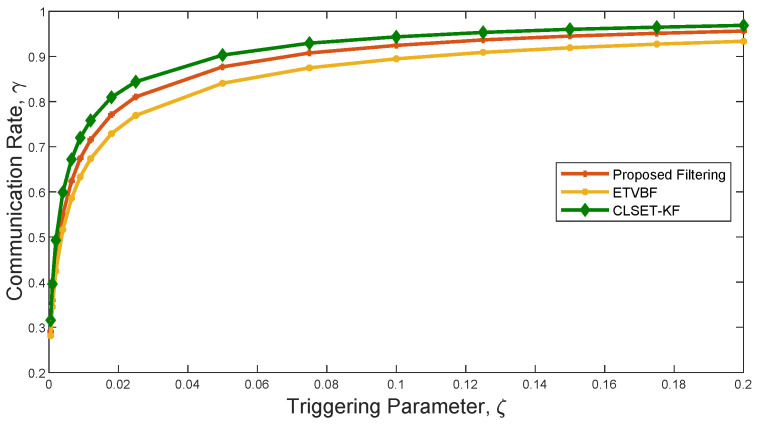
The communication rates of the proposed filtering, ETVBF, and CLSET-KF versus the triggering parameter.

Figure 3 shows the communication rate of the three filters using a closed-loop stochastic event-triggered scheme. As seen in Figure 2 and Figure 3, ETVBF has the lowest communication rate but the largest ARMSE. Compared with CLSET-KF, the proposed filter has both a smaller communication rate and better performance. Therefore, it can be concluded that the proposed filtering achieves a promising robust estimation performance with acceptable communication overhead under heavy-tailed noise.

**Figure 4:**
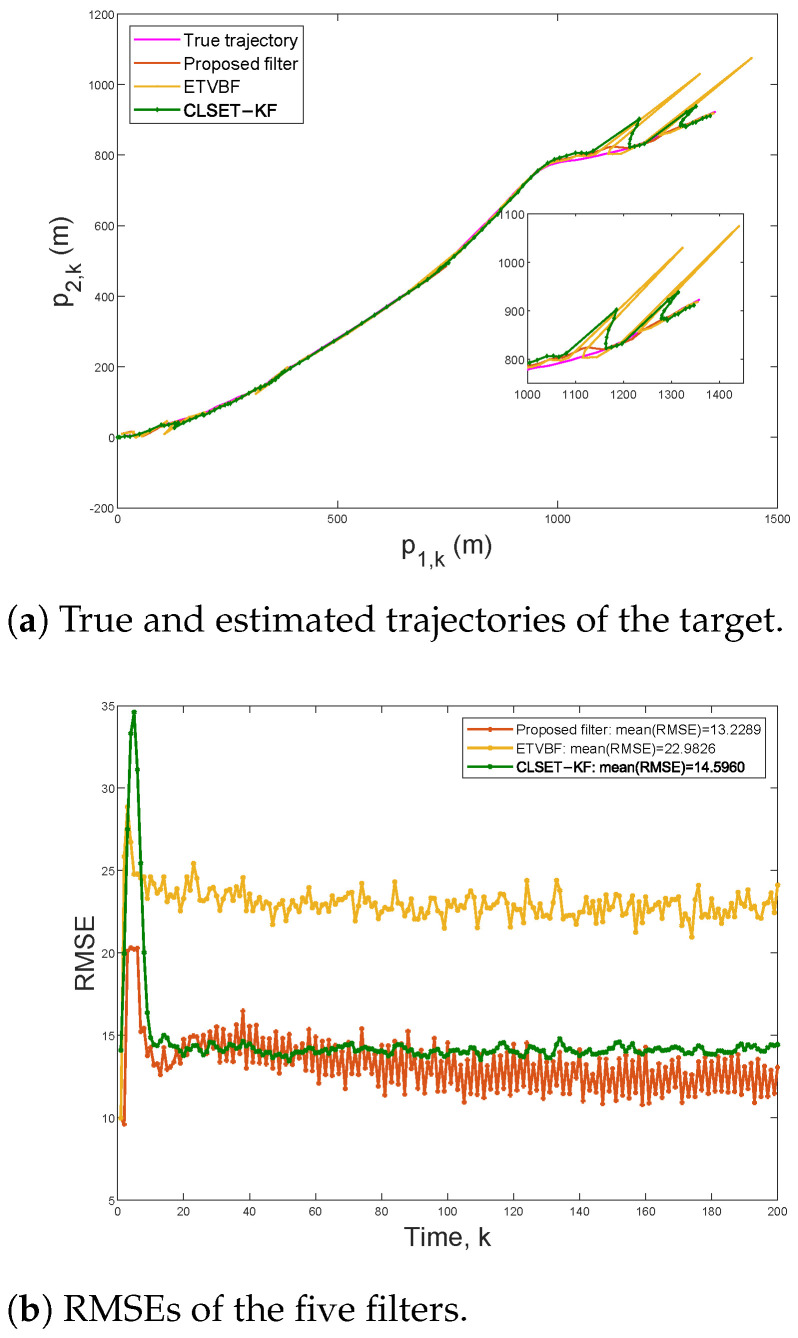
Trajectories and RMSEs under ζ=0.025 and β=100.

To compare the robust estimation performance under the stochastic event-triggered scheme, Figure 4 plots the target trajectories and the RMSEs within 200 steps under ζ=0.025 and β=100. In this case, the communication rates of the proposed filter, ETVBF, and CLSET-KF are 0.8108, 0.7695, and 0.8445, respectively. As shown in Figure 4a, ETVBF and CLSET-KF fail in tracking outliers, while the proposed filtering can still track the target position well. It can be seen in Figure 4b that the mean RMSEs of the proposed filtering and CLSET-KF are 13.2289 and 14.5960, respectively. As time step *k* increases, the RMSE of the proposed filtering is gradually and significantly smaller than that of CLSET-KF and ETVBF.

**Figure 5:**
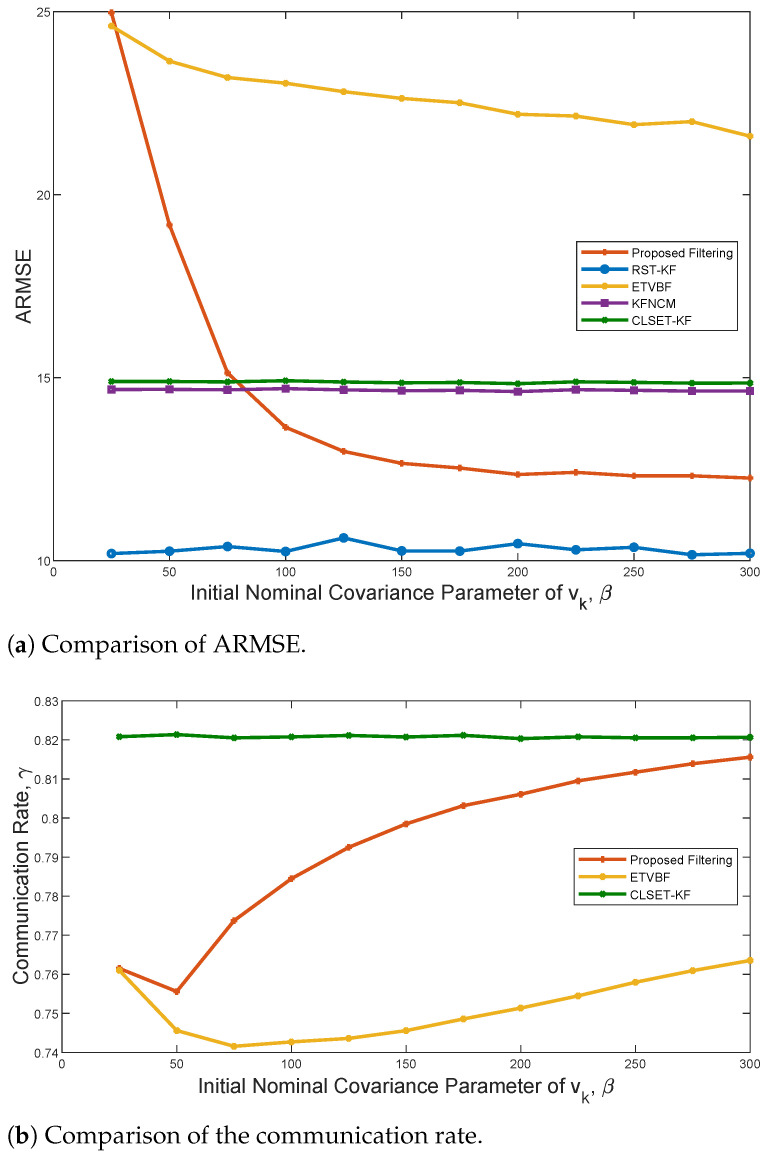
The comparisons of ARMSE and the communication rate versus β under ζ=0.02.

To illustrate the influence of the nominal measurement noise covariance ${\tilde{R}}_{0}$, the comparisons of ARMSE and the communication rate are depicted in Figure 5. Only ETVBF and the proposed filtering are affected by ${\tilde{R}}_{0}$, and both have better performance with increased β. It is also noted that the ARMSE of the proposed filtering tends to be stable when β is about 200. However, as β becomes large, the communication rate of both ETVBF and the proposed filtering decreases and then increases. Hence, a trade-off between the estimation performance and the communication rate can be achieved by adjusting parameter β.

## 6. Conclusions

In this paper, a robust variational Bayesian filtering method is proposed for the stochastic event-triggered state estimation system with heavy-tailed process and measurement noise. Heavy-tailed noise is approximated as Student’s t-distributions, where the prior distributions of the scale matrices are chosen as the inverse Wishart distributions. Based on a Student’s t-based hierarchical Gaussian state-space model, the variational Bayesian inference approach and the fixed-point iteration method are utilized to jointly estimate the system states and the unknown covariances in the cases of non-transmission and transmission, respectively. The simulation results demonstrate the effectiveness of the proposed stochastic event-triggered filtering method for heavy-tailed process and measurement noise with inaccurate covariance matrices.

This work was addresses the practical constraints of limited communication resources and outliers. Further work can involve the design of event-triggered robust state estimators for various other non-ideal implementations, such as communication delays, model uncertainties, and the extension of the proposed estimation method to real application scenarios.

## Data Availability

The original contributions presented in the study are included in the article.
